# Animal social learning: associations and adaptations

**DOI:** 10.12688/f1000research.7922.1

**Published:** 2016-08-31

**Authors:** Simon M. Reader

**Affiliations:** 1 Department of Biology, McGill University, Montreal, QC, Canada

**Keywords:** Animal social learning, social learning, adaptations, adaptationist, evolution, habituation

## Abstract

Social learning, learning from others, is a powerful process known to impact the success and survival of humans and non-human animals alike. Yet we understand little about the neurocognitive and other processes that underpin social learning. Social learning has often been assumed to involve specialized, derived cognitive processes that evolve and develop independently from other processes. However, this assumption is increasingly questioned, and evidence from a variety of organisms demonstrates that current, recent, and early life experience all predict the reliance on social information and thus can potentially explain variation in social learning as a result of experiential effects rather than evolved differences. General associative learning processes, rather than adaptive specializations, may underpin much social learning, as well as social learning strategies. Uncovering these distinctions is important to a variety of fields, for example by widening current views of the possible breadth and adaptive flexibility of social learning. Nonetheless, just like adaptationist evolutionary explanations, associationist explanations for social learning cannot be assumed, and empirical work is required to uncover the mechanisms involved and their impact on the efficacy of social learning. This work is being done, but more is needed. Current evidence suggests that much social learning may be based on ‘ordinary’ processes but with extraordinary consequences.

## Introduction

Animals learn from others. This phenomenon, termed ‘social learning’, is well established across numerous taxa and contexts, from fish learning mating sites by following others, to meerkats ( *Suricata suricatta*) teaching pups to handle scorpions ^1– 5^. Social information (information available due to the activities of other individuals) and social learning (learning from social information) can provide animals with a shortcut to adaptive behavior, minimizing the costs and risks of individual exploration ^6– 8^. For example, metabolic chamber studies demonstrate that socially acquired techniques result in considerable time and energy savings for black rats ( *Rattus rattus*) extracting seeds from pine cones and red squirrels ( *Tamiasciurus hudsonicus*) opening nuts ^9, 10^. Black rat pups learn the efficient cone stripping technique by stealing partially opened cones from their mothers, and the invention and spread of this technique has allowed black rats to invade newly planted pine forests in Israel, opening up a previously unexploited niche ^10^. As well as positive effects on animal success, several research groups have noted that the loss of socially learned behavior patterns may impact animal management and conservation (e.g. 11– 15).

Such examples illustrate the manifold ecological and evolutionary consequences that social learning can have, and a considerable body of theoretical work indicates that social learning will be advantageous in changing environments where genetic change is too slow and individual learning too costly to track change ^5^. However, social learning also raises the possibility of novel costs, such as increased competition over shared resources, or the acquisition of poor-quality or irrelevant information ^16, 17^. Animals have thus been proposed to employ so-called social learning ‘strategies’ or ‘biases’ that determine when to learn from others and who to learn from, increasing the payoffs of social learning ^6, 17, 18^. Such considerations of costs and benefits, together with the fact that social learning is a major contribution to the success of our own species, as well as observations of species differences in social learning, have led researchers to consider how, why, and when social learning and different social learning strategies have evolved. In this brief review, I discuss some current controversies within the field of social learning. Although research on human social learning is relevant to these controversies, much attention has been given to aspects of social learning thought to be uniquely human (e.g. 19– 23), and here I thus focus on non-human animals (henceforth ‘animals’). I also focus on the more general case of animals learning from cues inadvertently produced by the activities of other individuals, rather than learning from communication signals that are by definition evolved specializations ^24^.

## What evolves?

Social learning is defined in terms of its outcome rather than its underlying process. It can thus result from varied processes and mechanisms, and several classification schemes exist, often differentiating on the basis of the psychological processes thought to be involved, but also on the basis of what is learned ^5, 7, 25– 28^. Since multiple mechanisms may solve the same adaptive problem and multiple adaptive problems may be solved by a single mechanism ^29^, there need not be a one-to-one correspondence between social learning outcomes and mechanisms.

Considering how, why, and when social learning evolved has thus prompted researchers to ask, ‘what evolves?’ ^30, 31^. That is, what evolved processes underpin different instances of social learning, and have these evolved to facilitate social learning? More formally, have abilities to gather, assess, and utilize social information been specifically shaped by natural selection, resulting in derived adaptive specializations for social learning ^32^? Alternatively, is much social information use and social learning the product of general processes that have evolved or developed for other reasons? Or does most social learning instead result from a combination of these two possibilities? Social learning has often been assumed to involve at least some derived cognitive processes that evolve and develop independently, but this assumption is increasingly questioned.

A key counterargument to the adaptive specialization account has been the proposal that social learning propensities, strategies, biases, and processes are all products of general learning processes, with any adaptive specialization involving input systems rather than the learning mechanisms themselves ^7, 28, 31– 42^. Heyes has been key in developing and examining these ideas ^7, 28, 36, 41, 42^. For example, the recent papers ‘What’s social about social learning?’ and ‘Not-so-social learning strategies’ ^28, 42^ present considerable theory and evidence that social learning and social learning strategies depend on the same general learning mechanisms as individual (or ‘asocial’) learning. That is, while these learning mechanisms are themselves products of evolution, they have not evolved and are not specialized *for* social learning, nor have they subsequently been shaped by evolution to facilitate social learning. Instead, individual experience is argued to shape and specialize social learning.

Current, recent, and early life experience have all been shown to predict the reliance on social information ^43^, and thus experiential effects rather than evolved differences could indeed explain variation in social learning propensities between individuals, populations, and species, consistent with the general process account. For example, early maternal care predicts whether adult rats socially acquire food preferences ^44– 46^. However, flexibility alone is insufficient to rule out evolved social learning mechanisms, since flexibility could be genetically encoded. For example, individuals could follow evolved unlearned rules-of-thumb of when, where, and how to employ social information ^17^. Recent studies, in a variety of species but limited in number, have directly manipulated the value of social information. Such manipulations provide compelling evidence that social information use may indeed emerge as the result of within-lifetime learning rather than adaptive specializations ^31, 47^. For example, through simple associative learning, bees can be trained to approach but also to avoid flower colors that were previously marked by a social cue, just as they might learn the value of an asocial cue ^48^.

Data comparing individuals and species provide some further, albeit correlational, support for the idea that social learning is not independent from other processes ^49, 50^. For example, experimental tests of social learning and individual learning show that performance on these tests correlates across five species of birds, consistent with these traits evolving together ^32^. Similarly, in primates, the number of reported observations of social learning per species co-varies with both observational measures of behavioral flexibility and experimental measures of cognitive performance ^40, 51, 52^, although social learning may also carry specific costs in terms of parasite exposure ^52^. There is also comparative evidence consistent with evolved adaptive specializations in social learning. For example, Templeton *et al*. ^53^ found enhanced social learning in the more social of two corvid species over and above differences in individual learning, and human children were found to outperform two ape species on a variety of social measures, including social learning, while performance on physical tasks was more similar ^54^. Neither of the two study designs, however, fully eliminated developmental explanations or identified whether the specialization exists in learning mechanisms or input systems ^28^. A further issue relevant to all studies assessing reliance on social learning is the method of measurement. Typically, social learning is assessed as success or failure, or the speed of learning, but there are numerous additional ways to measure social learning performance, such as accuracy, longevity, generalization, number of demonstrations required to learn, the weight given to social information, the variety of acts acquired, and resistance to extinction. If these measures vary independently from one another or even negatively correlate (e.g. a speed-accuracy trade-off), ‘reliance on social learning’ may itself be a multidimensional trait.

Advantageous specializations in social learning could therefore be the result of genetic evolution, development, individual learning, or even social learning ^18, 41, 47, 55^. Once a bias to favor or disfavor social information has arisen, it may be strengthened by positive feedback during development, with individuals becoming more adept in particular types of social learning with experience ^50, 56^. While social and individual learning are often presented as alternatives, a view that has been criticized ^57^, a more fundamental distinction may lie in the degree that information is gathered for decision making, with certain individuals more likely to utilize both individual and social information ^58^.

## Do mechanisms matter?

As several scholars have noted (e.g. 28, 59), the neurocognitive mechanisms of social learning are woefully understudied, with some notable exceptions such as work on human fear learning and social influence (e.g. 60, 61), rodent food preference learning (e.g. 62), and birdsong acquisition (e.g. 63). This is clearly a problem for research orientated towards understanding mechanisms, such as work on human psychopathologies linked to social learning, but this gap also matters more broadly, for example to researchers focused on the adaptive function and evolution of social learning.

There are several reasons that research on the outcomes of social learning should also attend to the mechanisms of social learning (see also 42, 55). First, different mechanisms may have different transmission dynamics or fitness consequences (again a field of active debate ^57^). Second, if specializations in social learning do exist, they may allow valuable inferences to be made on the function of those processes, helping to establish the relevant costs and benefits ^64^. For example, uncovering evolved mechanisms that channel social learning to particular contexts or models would allow inferences to be drawn on when social cues provide useful information and when the attendant costs of competition are low. Third, knowing the mechanisms that underpin social learning allows us to determine what (if anything) has to evolve for social learning to occur and thus its likely distribution and impact. If much social learning is the result of general associative learning processes, as seems likely, this is exciting, since it widens the realm of both social learning and adaptive biases in social learning to any animal able to form learned associations.

When opportunities for learning about the value of social information are limited, learning or errors are costly, or the optimal response to a social cue is highly predictable, we would expect the evolution of genetically encoded predispositions that impact social learning, such as a bias to attend to particularly informative social cues (e.g. fear responses or feeding behavior). Biases and constraints impacting individual learning have been widely documented, and are proposed to dramatically increase the benefits of individual learning by facilitating the use of relevant cues and actions while allowing the many irrelevant ones likely to be present to go ignored ^65– 67^. Indeed, experimental evolution in *Drosophila* demonstrates that this ‘prepared learning’ about reliable cues can evolve readily ^67^. Similarly, work on animal communication has documented numerous adaptations in both signalers and receivers ^68^, demonstrating that adaptive specializations readily evolve in this domain too. The absence of evolved predispositions that impact learning from inadvertent social cues would thus be a great surprise, given the potential fitness payoffs of using this social information. If such predispositions are not found, it suggests that either flexibility is vital to adaptive function (e.g. social cues have variable meanings that must be learned) or the evolution of predispositions is constrained ^58^. The broad affordances of associative learning and its broad taxonomic distribution may mean that adaptive systems come with little additional cost, reducing the likelihood of alternate evolved solutions. For example, shoaling fish may learn about locations within their environment due to a tendency to group with and follow others combined with general learning abilities ^1, 69^, and thus in such cases the propensity to learn socially is intertwined with grouping propensities ^31, 70^. That is, grouping animals may get social learning benefits ‘for free’ as an exaptive by-product of forming groups ^71^. My view is that much variation in social learning can be explained as a result of experiential effects and general learning processes, or as a by-product of evolved changes in other traits, but that this will not be the whole story, and adaptive specializations that build upon pre-existing learning mechanisms are likely. These adaptive specializations may well be in input systems, but this does not make them unimportant.

## Concluding remarks

Both evolution and associative learning are powerful processes, and thus can potentially be used to explain many phenomena. Just as plausible but untested evolutionary explanations of traits have been criticized as adaptationist “just-so” stories ^72^, we must be cautious to avoid *associationist* “just-so” stories without empirical data and to ensure that underlying processes are carefully examined.

Associationist explanations are attractively parsimonious, since no new processes need evolve. However, without explicitly investigating processes, there is a danger of neglecting important specializations in input systems, for example, that may make particular associations more likely to be learned ^28^. Such specializations may be subtle but still have significant effects due to positive feedback processes, and how evolution and development interact to produce these specializations will have ramifications for the expected impact, flexibility, and taxonomic distribution of social learning. Identifying where, how, and whether specializations occur is challenging but worthwhile (e.g. 73, 74). Turning to general learning mechanisms, nonassociative learning processes such as habituation are proposed to underlie some instances of social learning, and thus should not be ruled out ^7^. Within associative learning, an open possibility is that certain domain-general parameters (such as the initial learning or extinction rate ^75^) are or have been shaped by the ubiquity, properties, or importance of social information in certain taxa. In sum, social learning depends on both social cues and on learning, and so we should not neglect the potential impact of processes outside of general associative learning mechanisms in shaping social learning propensities.

To conclude, this is a rich time for studies of social learning and social information use, with increasing work using novel experimental and mathematical methods to demonstrate the breadth of influence of social learning, often in large-scale studies of wild populations (e.g. 76– 80). Interdisciplinary integration has been key in this progress, and further integration between studies of mechanism and function provides exciting opportunities for new discoveries. Diverse fields thus have much to offer to our understanding of the causes and consequences of social learning.
